# AnyExpress: Integrated toolkit for analysis of cross-platform gene expression data using a fast interval matching algorithm

**DOI:** 10.1186/1471-2105-12-75

**Published:** 2011-03-17

**Authors:** Jihoon Kim, Kiltesh Patel, Hyunchul Jung, Winston P Kuo, Lucila Ohno-Machado

**Affiliations:** 1 Division of Biomedical Informatics, University of California, San Diego, CA, USA; 2 Bioinformatics Program, University of California, San Diego, CA, USA; 3 Laboratory for Innovative Translational Technologies, Harvard Medical School, Boston, MA, USA

## Abstract

**Background:**

Cross-platform analysis of gene express data requires multiple, intricate processes at different layers with various platforms. However, existing tools handle only a single platform and are not flexible enough to support custom changes, which arise from the new statistical methods, updated versions of reference data, and better platforms released every month or year. Current tools are so tightly coupled with reference information, such as reference genome, transcriptome database, and SNP, which are often erroneous or outdated, that the output results are incorrect and misleading.

**Results:**

We developed AnyExpress, a software package that combines cross-platform gene expression data using a fast interval-matching algorithm. Supported platforms include next-generation-sequencing technology, microarray, SAGE, MPSS, and more. Users can define custom target transcriptome database references for probe/read mapping in any species, as well as criteria to remove undesirable probes/reads.

AnyExpress offers scalable processing features such as binding, normalization, and summarization that are not present in existing software tools.

As a case study, we applied AnyExpress to published Affymetrix microarray and Illumina NGS RNA-Seq data from human kidney and liver. The mean of within-platform correlation coefficient was 0.98 for within-platform samples in kidney and liver, respectively. The mean of cross-platform correlation coefficients was 0.73. These results confirmed those of the original and secondary studies. Applying filtering produced higher agreement between microarray and NGS, according to an agreement index calculated from differentially expressed genes.

**Conclusion:**

AnyExpress can combine cross-platform gene expression data, process data from both open- and closed-platforms, select a custom target reference, filter out undesirable probes or reads based on custom-defined biological features, and perform quantile-normalization with a large number of microarray samples. AnyExpress is fast, comprehensive, flexible, and freely available at http://anyexpress.sourceforge.net.

## Background

With rapid accumulation of gene expression data in public repositories such as NCBI GEO[1], integrated analysis of multiple studies is receiving increased attention. The integrated analysis increases statistical power, generalizability, and reliability, while decreasing the cost of analysis, since it exploits publicly available data for related studies, which are often from different platforms [2,3]. Rhodes *et al. *identified a set of differentially expressed genes between prostate cancer patients and healthy subjects from an integrated study of four different datasets and discovered that some genes were consistently dysregulated in prostate cancer but were not reported in the individual studies [4]. Warnat's group performed a classification study of cancer patients with six different datasets and achieved higher accuracy over single-set analysis [5]. Both studies were conducted across different gene expression platforms.

Despite the well-known benefits, conducting a cross-platform analysis of gene expression data involves many intricate issues at different layers. A recent guideline discussed several key issues when conducting an integrated microarray data analysis: annotating probes of the individual dataset, resolving the many-to-many relationship between probes and genes, aggregating multiple measurements into a single gene-level value, and combining study-specific estimates [2]. Some authors noted that the interpretation of the biological results could be improved with re-annotated and filtered probes in microarray studies [6,7]. These probes would ideally be at no risk of cross-hybridization to multiple genes and would not contain any SNPs or repeats in its sequence [8,9].

Several tools were developed to resolve the aforementioned hurdles for cross-platform analysis of gene expression data. CrossChip http://www.crosschip.org provides comparative analysis between different generations of Affymetrix arrays [10]. It utilizes architectural information of probe, i.e., the minimum sequence overlap length and the minimum probe pairs per probe-set to enable cross-platform comparison, but the scope is limited to Affymetrix platforms. Another tool, EXALT http://seq.mc.vanderbilt.edu/exalt, allows the user to upload his/her data and searches for homologous data sets obtained from public repositories. However, the user is still responsible for ascertaining the quality of the probe and its impact at the gene expression level [11]. Furthermore, neither of these tools takes into account the biological characteristics of probes, such as presence of SNPs or repeat sequences. EXALT recommends the use of GDS (processed data by NCBI)--pre-processed data derived from GSE (raw data submitted by authors)--but the derived measurement values are problematic as they still contain undesirable probes that map to multiple genes, are specific to a certain transcript, or may contain a SNP. Furthermore, only a fraction of all studies in GEO have a corresponding GDS. In our recent study of 58,432 GEO microarray samples from six different diseases, only 19.7% of them were included in GDS [12]. Another available tool, A-MADMAN http://compgen.bio.unipd.it/bioinfo/amadman, performs integration of cross-platform microarray data obtained from GEO [13]. However, its input is limited to Affymetrix platform microarrays and the probe annotation relies on available chip description files, which are known to have errors or are outdated, as biological knowledge is updated [6,7]. CPTRA http://people.tamu.edu/~syuan/cptra/cptra.html, another tool for cross-platform analysis of gene expression data [14], allows two different platforms to be combined, but the focus of this software is on the species with limited genome information, such as horseweed [14]. Hence, one of the inputs must be a long-read sequence with proper annotation. In contrast to CPTRA, our analysis tool, AnyExpress focuses on well-studied species like human, mouse, fruitfly or Arabidopsis, where reference genome information and the transcriptome database are well-maintained and available.

Our approach is to start the analysis from raw files, such as .*fastq *(Roche 454 or Illumina GA), .*csfastq *(ABI SOLiD color-space), or .*fasta *(microarray platforms, SAGE or MPSS) to remove undesirable probes *before *pre-processing. For example, we summarize multiple probe level measurements into a single target-level value, where the target is a user-defined expression unit (e.g., gene/isoform/exon). None of the existing tools can handle integration of NGS and microarray data from different platforms. Thus, we developed a practical, integrated toolkit for cross-platform analysis of gene expression data serving all NGS and microarray platforms for any species. Previously, our group demonstrated that sequence-based probe matching improved the consistency of measurements across different platforms, compared to the widely-used identity-based matching method at that time[15-17]. We also developed DSGeo, a software collection for analyzing microarray data deposited in GEO [18]. We now extend our previous work, by integrating a novel interval-matching algorithm [18-20] and developing a suite of software tools, called AnyExpress. Our suite of tools automates the matching of NGS, microarray, SAGE and MPSS, and also allows users to define reference target and probe quality filters.

## Implementation

### Architecture

**Figure 1:**
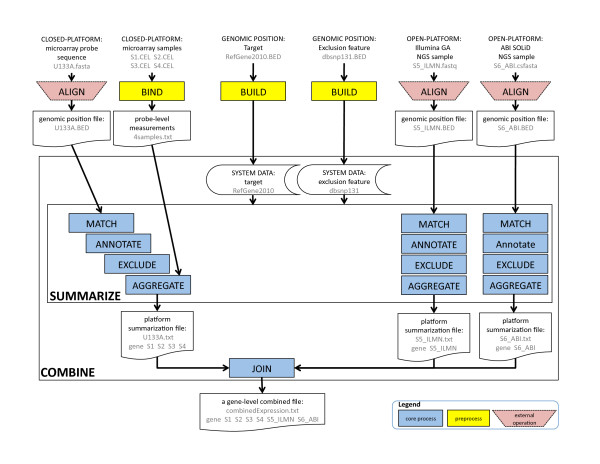
**Workflow of AnyExpress**. An outline of data flow is depicted with input, output, and intermediate files. Core processes in AnyExpress are drawn as blue rectangles. Pre-processing is represented by yellow rectangles. ALIGN is an external process (pink trapezoid) that runs via software such as Bowtie or RMAP. The standard input to AnyExpress is a Browser Extensible Data (BED) file or a tab-delimited multi-column file. The output is a target-by-sample combined file. A gene-by-sample is used in the final output of this figure, but the user can choose his/her own target (e.g., 'RefGene isoform' or 'Ensembl gene') by running *anyexpress Build*.

AnyExpress is a software suite for cross-platform analysis of gene expression data. It allows two sources of inputs: (i) genomic position files, obtained from the external alignment software and (ii) probe-level sample measurements files. AnyExpress returns a target-by-sample text file as an output. We define 'tag' as a string of nucleotide sequences used for measuring gene expression abundance. This string is commonly called 'probe' or 'read' for microarray or NGS platform, respectively. Throughout this article, we use tag, probe, and read interchangeably. Next we define 'platform' as a set of tags. Then, we classify platforms into one of two classes, based on the availability of knowledge in a tag's sequence. When the tag sequence was predetermined, as in a microarray or catalysed reporter deposition (CARD) FISH, the platform was considered to be *closed-platform *[21,22]. If the tag sequence is determined at the time of sequencing, as it is in NGS, serial analysis of gene sequence (SAGE), or differential display (DD), the platform is considered to be *open-platform *[21,22]. While closed-platform can have multiple samples (e.g., 20.*cel *files of the same platform, an Affymetrix U133A microarray), the open-platform has a 1-to-1 relationship with the sample (e.g., six Illumina GA NGS .*fastq *files from six corresponding patients). AnyExpress is capable of dealing with gene expression data from all platforms in contrast to the existing tools that focus on a single platform. A schematic workflow of AnyExpress is displayed in Figure 1. The gene expression data of one closed-platform (Affymetrix U133A) and two open-platforms (Illumina GA and ABI SOLiD) are combined. A summarization file is created per platform as a result of the SUMMARIZE process, then the multiple summarization files are merged into a single gene-by-sample text file through a JOIN process within a COMBINE process. Before running core processes of AnyExpress (shown as blue rectangles), the user needs to build target and reference features (indicated by yellow rectangles) to generate 'SYSTEM DATA' and manually perform sequence alignment using external software tools such as Bowtie (indicated by pink trapezoids) [23].

### Reference target

We refer to a *target *as a biologically meaningful expression unit against which tag will be matched using genomic positions. Each target is a collection of five attributes: chromosome, strand, start position, end position, and identifier. AnyExpress accepts the target as a .*BED *file where the five fields are separated by tabs. In most cases a single target has multiple associated tags; hence, multiple measurement values will be summarized into a single aggregated value. The target identifier must consist of two substrings concatenated by '@', i.e., targetID = 'superID' + '@' + 'subID'. For 'BRCA1' gene, its identifier (with the corresponding target) could be represented as 'BRCA1@Exon2' (official gene symbol), 'NC_007294@Exon2' (RefSeq), or 'ENSG00000012048@Exon2' (Ensembl). The target information will be updated as knowledge of the genome and genes evolve. Species, source database, and build-date are three factors defining a target. Example of .*BED *format files are as following: 'Human_Ensembl_Feb2009.BED,' 'Human_UCSCKnownGene_Feb2009.BED,' 'Human_RefSeq_Feb2009,' and 'Human_RefSeq_Mar2006.BED'. Unlike existing custom reannotation data approaches in which the user's analysis is limited by a particular type of target that the annotator has predefined, AnyExpress allows users to define their own reference target for any species. In Figure 1, RefGene2010 was selected as a target and the corresponding system data was created by running *Anyexpress Build* before running *Anyexpress Combine* all in a command-line.

### Exclusion features to identify undesirable tags

Exclusion features allow users to apply a biological filter applied against the tags to filter out undesirable ones. Previous studies have shown the negative effect of low quality microarray probes on measurement of gene expression abundance and consequently on the interpretation of the results [6,8,9,24,25]. A probe that hybridizes to more than one reference target is referred to as a 'cross-hybridization' or 'multi-target' probe. This type of probe often results in ambiguous interpretation of results, negatively affecting downstream analysis such as statistical testing, clustering, or enrichment analysis on Gene Ontology or pathways [6,25]. The presence of SNPs within the probe sequence would cause incorrect estimation of mRNA abundance [6,8]. It has been reported that the removal of undesirable tags resulted in increased statistical power to detect differentially expressed genes [9,25]. Existing tools or custom CDF files restrict users to a predefined set of filters, sources, and build dates according to external annotators [9,26]. For example, a SNP alone can have several characteristics: class of variant (single, in-del, or unknown), functional category (coding-synonymous, intron, noncoding-synonymous, near-3', near-5', or unknown), validation status (by-cluster, by-frequency, by-hapmap, or unknown), and average heterozygosity [24]. AnyExpress offers flexible solutions where the user can define desired characteristics and selectively apply tag-filters at the time of data integration.

### Interval matching algorithm

AnyExpress takes the outputs of external alignment software as inputs (e.g., Bowtie for NGS), which consist of a list of attributes of genomic position (chromosome, strand, start, and end). Probes and reads are matched against targets. Matching two entities based their genomic positions is a core part of data integration process in AnyExpress. While naïve comparison of all intervals of target and tag (e.g., RefSeq vs. NGS read) is a computationally-intensive task with time complexity *O*(*n^2^*), AnyExpress adopts a fast interval matching algorithm called PositionMatcher, developed by our group [20]. PositionMatcher performs "genomic walking" by iterating linearly along the positional stamp of a genome, keeping track of overlapping intervals in a hybrid data structure of stack and queue to achieve time complexity down to *O*(*nlogn + n*). In a previous study [20], we showed that the execution time of PositionMatcher was over 20 times faster than that of all-pairwise comparison methods using the Illumina NGS data reported in Marioni *et al.*[27] as an example.

**Figure 2:**
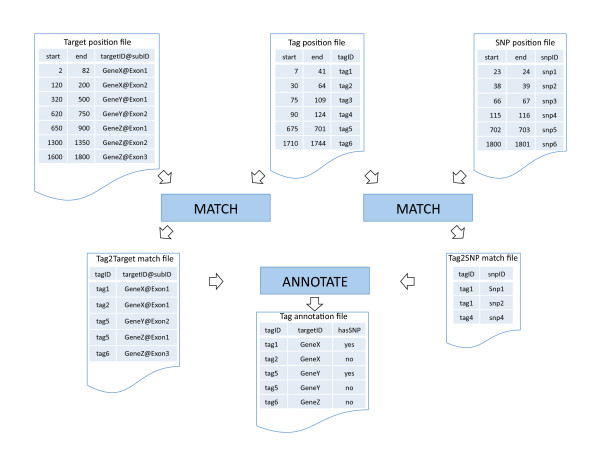
**Illustration of two processes: MATCH and ANNOTATE**. The MATCH process takes as input two position files (each with three columns: start, end, and identifier) and generates as an output, a list of ID pairs, tag ID, and target ID. While MATCH on the left was between tag and target, MATCH on the right occurred between tag and SNP. Then ANNOTATE merged the two outputs of MATCH to create a tag annotation file with associated target ID and indication of SNP presence/absence.

Figure 2 shows an example of how MATCH process is performed: the first match relates tag to target and the second match relates tag to SNP. The result of matching is a list of two objects, (target, tag) or (target, SNP). Then, ANNOTATE process generates a tag annotation file to report each of the tag's associated targets and the presence/absence of SNPs in the sequence. Based on this annotation file, EXCLUDE and AGGREGATE processes produce a summarization file for each platform to feed into JOIN process (Figure 1).

### Platform-level summarization

As illustrated in Figure 1, the output of SUMMARIZE process, per platform, is a target-by-measurement value text file where multiple measurements are aggregated into a single numeric value per target. For a closed-platform, multiple tag-level signals are summarized into a single number per target-sample pair. We used Tukey's median-polish algorithm, a widely used summarization technique in microarray data, to introduce the robust multi-array averaging (RMA) method [28]. For an open-platform, multiple associated tags were aggregated into a single 'Reads Per Kilobase exon Model per million mapped reads' (RPKM) value per tag [29].

### Auxiliary tools with high scalability to create input data

In early microarray studies, the number of samples for Affymetrix was small (less than 20), so it was easy to create a single column-bound file. But recent studies involve a large number of samples, often exceeding 200, which makes data loading impossible using R, Matlab, or stand-alone software due to limited memory size. Although the number of tags is relatively lower in microarrays than in NGS (1 million versus tens of millions), currently, the number of samples is larger due to the maturity and inexpensive cost of this technology. For example, 186 Affymetrix microarray *.cel *files were used in a lung cancer classification study [30] and 286 *.cel *files were used in a breast cancer study [31]. These individual studies are already large and the integrated analysis incorporating those will evidently be even larger. Simple loading of individual *.cel *files using conventional computers fails even before normalization or summarization. Solutions using parallel computing are being proposed, but these are useful only to users with advanced skills and access to high performance computing resources [32]. Thus, we propose AnyExpress as a solution. It is developed to serve the average user, one that has access to 4 ~ 8 GB of memory and a 2 ~ 3 GHz processor.

#### Binding a large number of Affymetrix .cel files

We defined the input format of closed-platform samples for AnyExpress as a single column-bound, tab-delimited text file where the first column is a probe identifier followed by measurement values of the samples in the second column. This is a common data format for microarrays in non-Affymetrix platforms. However, in Affymetrix, each sample is a .*cel *file and needs to get column-bound. AnyExpress provides a scalable binding tool, *anyexpress BindAffyCel* (in a command-line), to create this single column-bound file from a large number of Affymetrix .*cel *files. We tested the capability of AnyExpress in binding a different number of .*cel *files downloaded from GEO. For binding, AnyExpress extracts probe identifier as probeID = 'x-coordinate' + ':' + 'y-coordinate' from the .*cel *file. The user is required to place .*cel *files of the same platform in the same directory. Only the probe identifiers that are common across all samples will be represented in an output file. In Figure 1 (top left), four Affymetrix .*cel *files are bound to a single text file '4samples.txt'.

#### Quantile-normalization for a column-bound data of microarray samples

Quantile-normalization [28] is a widely used pre-processing procedure for microarray data, but its processing is severely limited by certain hardware. The column-bound file can be directly used in *anyexpress Combine*, but it is highly recommended to perform between-sample normalization of this data to remove systematic bias to enable fair comparison among samples [13,33]. Of the different normalization algorithms, the quantile-normalization was shown to be superior [13,33]. Quantile-normalization is a rank-invariant transformation of measurement values that have identical distribution of measurement values across all samples once they get processed. The same scalability issue applies to quantile-normalization. A single .*cel *file contains over 500,000 probe-level measurement values. When hundreds of samples need to be combined, existing tools can hardly perform quantile-normalization. AnyExpress solves this issue with a highly scalable tool, *anyexpress NormalizeColumnBoundSamples*.

### Coverage plot

Visualization plays a critical role in data validation, interpretation, and hypothesis generation during analysis [34]. Software tools for visualization should be able to manage a large number (e.g., millions) of tags. We developed a tool that can create a coverage plot along the genome for all the platforms used in a single AnyExpress run. The output file is a .*bedGraph *text file. The user needs to upload this file onto the UCSC Genome Browser http://genome.ucsc.edu through his/her own web-browser. The user can draw a plot by typing in five parameters: a directory of user, *Project*; chromosome; strand ('forward' or 'backward'); start position; and end position. Each platform, closed or open, in the user's *Project *is drawn as a track in the *.bedGraph *file. In each track, vertical bars are drawn along the genomic region of interest. The height of the vertical bar is either the number of the reads covering each base in open-platforms or the average signal intensity of the probe covering each base. As a default reference track, the RefSeq gene model is displayed at the bottom of the plot. The user can freely add, hide, or modify the plot through the UCSC Genome Browser, e.g., adjust scales, change color, or add biological reference tracks.

### Operation

**Figure 3:**
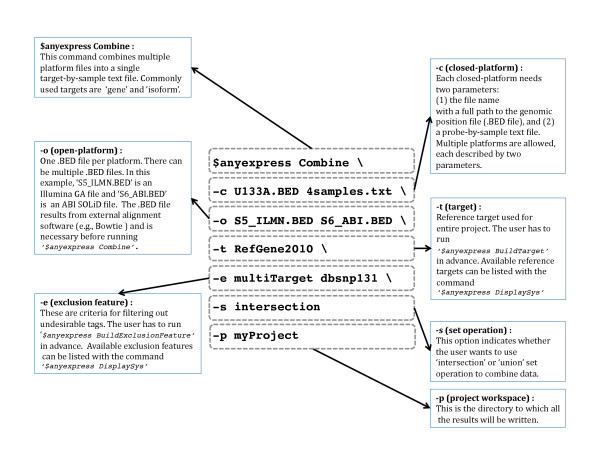
**Command line example**. A command line to execute AnyExpress Combine is illustrated. The command can run in any Unix-like environment or MS-DOS in Windows. A backslash ('\') character is used to continue the command onto the next line. All .*BED *and .*txt *files are assumed to be located in the current working directory. If they are not, the full path should be used. Also, the target 'RefGene_2010 and two exclusion features 'multiTarget' and 'dbsnp131' are assumed to be created before running *anyexpress Combine*.

AnyExpress is composed of an executable wrapper (shell script or .*exe *file), a collection of Java classes and pre-processed data (reference targets and exclusion features). Once an archived file (.zip) is extracted to the user's local machine, AnyExpress is ready to execute after ENVIRONMENT and PATH variables are set, as in any other command-line software for a Unix-like environment or Windows. Tools available in AnyExpress are summarized in Table 1. Instructions on installation, configuration, and usage are detailed in the accompanying webpage http://anyexpress.sourceforge.net. Among seven tools, COMBINE is the main process that performs data integration. Figure 3 explains the option parameters by showing an example of running *anyexpress Combine* in a command-line, to combine closed-platform data (microarray: Affymetrix U133A) and two open-platform data sets (NGS: Illumina GA and ABI SOLiD). Tags from these three platforms were matched against 'RefGene2010' using the PositionMatcher algorithm. The tags were also matched against targets 'multiTarget' and 'dbsnp131' for filtering. The exclusion feature 'multiTarget' is automatically generated during the ANNOTATE process. For example, in Figure 2, tag5 is matched to two genes, geneY and geneZ (bottom left table in Figure 2). Once such tag-to-target pairs are obtained, a 'multiTarget.txt' file that contains a list of undesirable tags, such tag5 in Figure 2, is created. The final output 'combinedExpression.txt' is created under the user-specified directory (specified as 'myProject' in Figure 3) and also contains summary statistics.

**Table 1:** Summary of AnyExpress tools

TOOL	DESCRIPTION
*BindAffyCel*	Binds multiple Affymetrix microarray .*cel *files column-wise into a single probe-by-sample text file
*BuildExclusionFeature*	Creates exclusion features for filtering out undesirable tags
*BuildTarget*	Creates reference targets for matching tag positions, using the user-selected transcriptome database
*Combine*	Combines both open- and closed-platform gene expression data into a single target-by-sample text file
*DisplaySys*	Prints currently available reference targets and exclusion features in the system directory
*NormalizeColumnBoundSamples*	Performs quantile-normalization on a probe-by-sample text file
*Plot*	Creates a coverage plot along the genomic region (.*bedGraph *format), which needs to be uploaded to the UCSC Genome Browser for viewing

### Tested platforms

AnyExpress was implemented in Java, shell script, and Python and it runs on Unix, Linux, Mac OS X, and MS-DOS in Windows. AnyExpress successfully worked with three different configurations: (i) a 64-bit Linux server with a 2.13 GHz Intel Core™ 2 Duo CPU and 16 GB memory, (ii) a 32-bit iMac with a 2.66 GHz Dual-core Intel Zion and 4 GB memory, and (iii) a 32-bit Windows 7 with a 1.8 GHz Intel Core CPU and 4 GB memory. The executables, the source code, the example data, and the manual are available at http://anyexpress.sourceforge.net.

## Results

### Combining NGS and microarray data

We applied AnyExpress to human gene expression data from Marioni *et al. *[27]. It consists of six microarray samples (Affymetrix HG U133A) and six Illumina GA NGS samples. We downloaded raw microarray .*cel *files from the NCBI GEO database (accession number: GSE11045) and raw NGS .*fastq *files from NCBI Sequence Read Archive (submission number: SRP000225). The six samples, all human tissue, were obtained from kidney (3 samples) and liver (3 samples). For pre-processing, six microarray .*cel *files were bound into a single column-bound file after running AnyExpress:

*$anyexpress BindAffyCel ~/celfiles 6sample.txt*

To create a closed-platform .*BED *file, we downloaded a probe sequence file (.*fasta*) of Affymetrix U133 Plus2 from the Affymetrix Support webpage http://www.affymetrix.com and processed it to have a probe identifier as in 'x coordinate' + ':' + 'y-coordinate', used by Thompson *et al. *[25]. Then we aligned the probe against the genome sequence to obtain genomic positions for the probes using two external tools, Bowtie [23] and AWK [35]:

*$ bowtie ~/indexes/hg19 -t -n 0 -B 1 hg19 -f U133PLUS2.fasta U133PL2.bowtie*

*$ awk '{ FS="\t"; OFS="\t"; print $3, $4, $4+length($5)-1,\ $1, $2 }' U133PLUS2.bowtie > U133PLUS2.BED*

Bowtie is a fast and memory-efficient algorithm and tool for short sequence alignment [23] and AWK is a convenient Unix-like environment tool for processing a text file [35]. We chose these tools for their popularity and convenience, but users can freely use other tools or their own code to process their .*fasta *files to obtain the .*BED *format file. For Windows users, we provide an AWK-equivalent tool, awk.exe, through the AnyExpress webpage. Open-platform files were aligned and processed in the same way as closed-platform files. The only difference was to replace the Bowtie option from '*-f*' to '*-q*' because NGS data used the *.fastq *format. The following Bowtie-awk running was repeated for all six NGS files (SRR002320.*fastq *through SRR002325.*fastq*):

*$ bowtie -t -n 0 ~/indexes/hg19 -q SRR002320.fastq SRR002320.bowtie*

*$ awk 'BEGIN {FS = "\t"; OFS="\t"} {print $3, $4, $4+length($5)-1,*

*$1, $2 }' SRR002320.bowtie > SRR002320.BED*

We built target and an exclusion features into the system using AnyExpress:

*$ anyexpress BuildTarget RefSeq_Gene.BED*

*$ anyexpress BuildExclusionFeature dbsnp131.BED*

The resulting files were all created in the *'$ANYEXPRESS_HOME/sys/target' *and *'$ANYEXPRESS_HOME/sys/exclusionFeature' *directories.

We combined all 7 platforms (1 closed-platform + 6 open-platforms) of the Marioni data with AnyExpress, using multiTarget and dbsnp131 as exclusion features:

*$anyexpress Combine -c U133PLUS2.BED 6samples.txt -o SRR002320.BED SRR002321.BED SRR002322.BED SRR002323.BED SRR002324.BED SRR002325.BED -t RefSeq_Gene -e multiTarget dbsnp131 -p /user/jkim/myProject*

**Figure 4:**
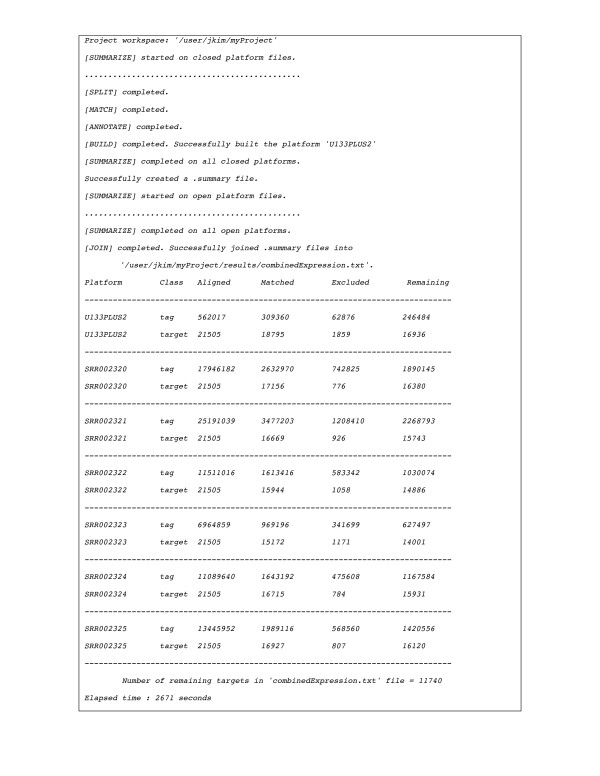
**AnyExpress run-time message log**. Run-time messages are shown during the analyses, with statistics and execution times shown at the bottom.

Run-time messages during the AnyExpress execution are shown in Figure 4. The start and end of tasks are displayed in a step-by-step manner. The final combined file is a target-by-sample text file that can be used in downstream analyses, such as identification of differentially expressed genes, classification, clustering or enrichment analysis on Gene Ontology and pathways [36]. At the bottom of Figure 4, coverage statistics of tag and target are added along with the execution time (2,671 seconds).

We calculated Spearman correlation coefficients (CC) for the combined data to assess reproducibility. The within-platform CCs were very high in both kidney and liver (mean CC = 0.980; sd CC = 0.011), while cross-platform CCs were moderate (mean CC = 0.733; sd CC = 0.001). These results confirmed the results of the original study [27]. The cross-platform CC = 0.733 is similar to our previous results for cross-platform microarray studies [16,17] and similar to results from the MAQC consortium [17]. Although we observed lower cross-platform CCs, it is known that a decrease in correlation could be due to tag-effect differences in each platform [37].

For visualization, we drew a coverage plot in the genome regions of gene GPX3 (chr5:150,395,999 - 150,410,551):

$anyexpress Plot/user/jkim/myProject chr5 forward 150395999 150410551

**Figure 5:**
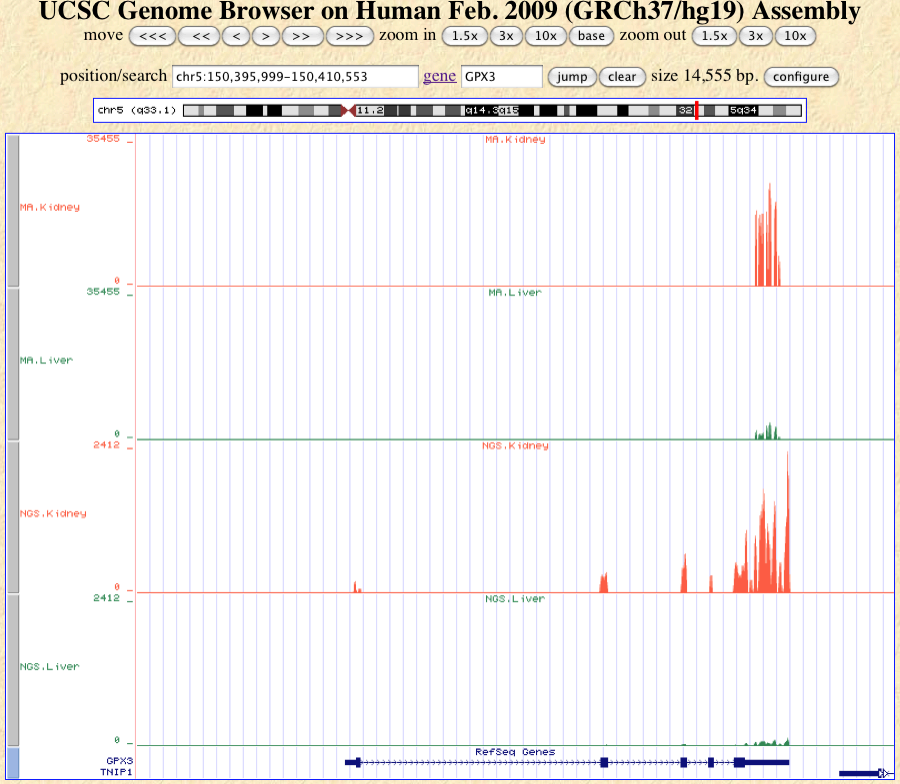
**Coverage plot**. An example of a coverage plot generated from the *anyexpress Plot* module in the human genomic region of the GPX3 gene (from 150,395,999 to 150,410,551 in chr5). The top two tracks represent kidney (red) and liver (green) samples measured by microarrays (MA). The bottom two tracks represent kidney (red) and liver (green) measured by NGS. Height rescaling and color changes were applied in the UCSC Genome Browser for a better view.

This gene is shown to have tissue-specific expression, higher in kidney but lower in liver [38]. The resulting *.bedGraph *file was uploaded to a custom track of the UCSC Genome Browser for visualization. We selected four representative tracks out of the original twelve due to page limitations and adjusted the browser setting for clearer viewing. Figure 5 displays the difference between the two technologies. As expected from Affymetrix's original probe design scheme, microarray probes were only found in the last exon. In contrast, Illumina GA reads spread across all exons. Figure 5 demonstrates that differential expression between two tissues, kidney (red) vs. liver (green), is well-conserved within each platform.

### Effect of exclusion features

**Figure 6:**
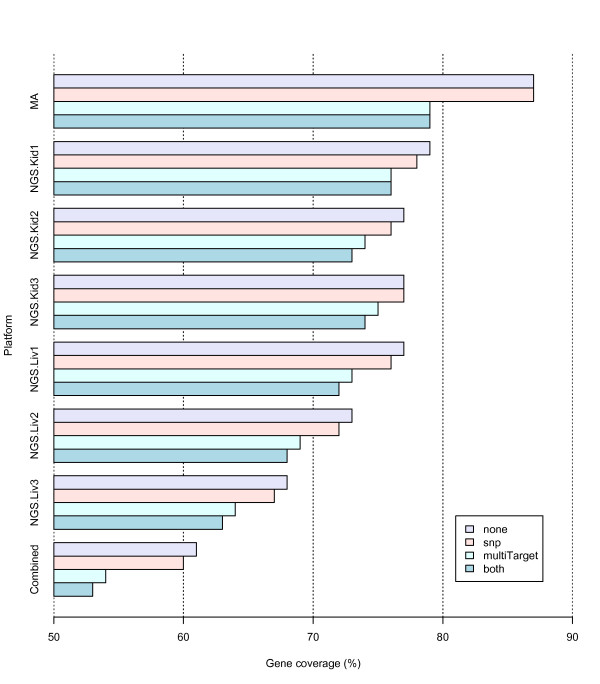
**Gene coverage**. Gene coverage per platform is displayed with different exclusion feature settings. The coverage was calculated using the RefSeq transcriptome database as a reference, which had a total of 21,505 genes. Each platform had 4 coverage values obtained from the corresponding exclusion features: 'none' = apply no exclusion feature, 'snp'= remove tags containing SNPs, 'multiTarget' = remove tags matched to more than one target, and 'both' = apply both 'snp' and 'multiTarget'. Both microarray (MA) and next-generation sequencing (NGS) had replicates from two tissues: kidney (Kid) and liver (Liv). The coverage that resulted from applying all filters is shown at the bottom of the graph (Combined).

We ran AnyExpress on the Marioni data with four different exclusion feature settings: 'none' = apply no exclusion feature, 'snp'= remove SNP containing tags, 'multiTarget' = remove tags matched to more than one target, and 'both' = apply both 'snp' and 'multiTarget'. We assessed the effect of exclusion features on gene coverage and correspondence of highly expressed genes across the platforms. Figure 6 displays gene coverage of seven platforms of microarray (MA), six NGS (NGS.*) and the final combined expression (Combined). The coverage was calculated as the number of genes that remained after filtering divided by the total number of genes in the RefSeq transcriptome database (total = 21,505). Microarray had the highest coverage value and the combined file had the lowest since it only keeps genes from the intersection of the other six platforms. (AnyExpress also allows 'union' as a set operation.) Application of exclusion features resulted in slightly lower coverage per platform. Within NGS, overall coverage was higher in kidney (Kid) than in liver (Liv).

**Figure 7:**
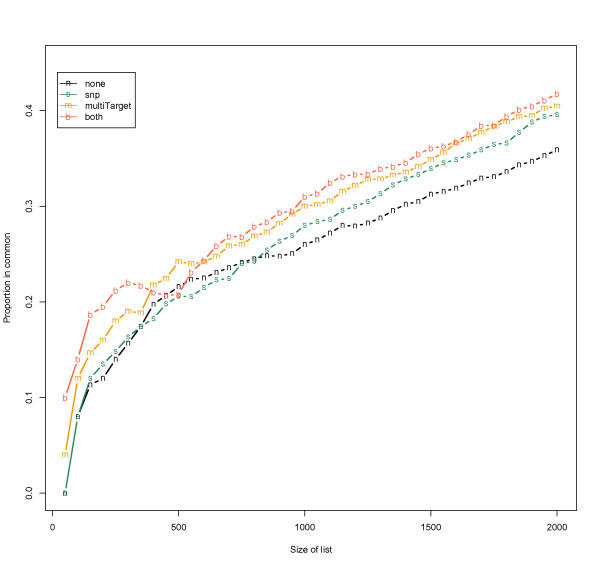
**CAT plot using NGS as reference**. This CAT plot depicts the agreement between NGS and microarray data on the detection of differently expressed genes. The X-axis is the number of top genes in NGS data, ranked by log2 fold-changes of kidney vs. liver. The Y-axis is the proportion of genes from the microarray that is in common with top-ranked genes from NGS. Four CAT plots are drawn using none, snp, multiTarget, and both (snp and multiTarget simultaneously) exclusion features.

In Figure 7, cross-platform agreement for highly expressed genes is assessed with the correspondence at the top (CAT) plot, first introduced by Irizarry *et al. *[37]. Correlation coefficients were shown to be inadequate to assess correspondence between studies or platforms, due to a small number of differentially expressed genes [3]. Hence, other authors have suggested that cross-platform agreement should be evaluated on genes which are likely to be differentially expressed [3,37]. Previously we used this plot in a cross-platform study of microarray and MPSS [16]. The CAT plot has also been used in several similar studies [3,39,40]. We created lists of highly expressed genes, size *n*, sorted by fold-change in decreasing order, varying *n *from 50 to 2000 by 50. For each top-*n *genes from NGS, we counted the number of genes that were in common with the top-*n *genes from microarray and divided this number by *n *. As expected, the proportion that was in common between the two platforms increased with an increase in *n*. The agreement proportions in 'none' and 'snp' were similar when the list size was smaller than 900, but the proportion was higher in 'snp' than in 'none' when the list size was above 900. 'multiTarget' and 'both' outperformed 'none' for all list sizes. Overall, the CAT plot demonstrated that filtering by exclusion features produced higher agreement between the two platforms. We also assessed the cross-platform correspondence with a modified CAT plot where genes were ranked by a false discovery rate (FDR) adjusted q-value [41], instead of fold-change (Additional file 1). 'snp' and 'none' showed similar correspondence, but overall we observed the same effect of larger correspondence with filtering.

### Execution time with a large number microarray samples

We performed stress testing of AnyExpress with a different number of .*cel *files under different memory sizes (Table 2). The number of .*cel *files was increased per memory size until failure (i.e., encounter of memory allocation error). Pre-processing processes (BIND or NORMALIZE) took longer than the actual COMBINE process. We found that AnyExpress can manage up to 500 .*cel *files with 8 GB memory. The user needs to have a memory size larger than 8 GB to process more than 500 .*cel *files. At the time of writing this manuscript, the price of a 4 GB memory was around 100 US dollars. Considering the cost of high-performance computing, running AnyExpress with additional memory on an average PC or laptop computer is cost-effective for a large-scale cross-platform analysis of gene expression data.

**Table 2:** Stress testing with Affymetrix .*cel *files

Allocated Memory (GB)	Number of Affymetrix *.cel *files	Execution Time (seconds)		
		Bind	Normalize	Combine
4	100	245	253	78
	200	1057	885	252

5	100	230	257	68
	200	668	582	165
	300	1355	1084	296

6	100	222	249	68
	200	660	578	167
	300	1312	1053	289
	400	2207	1515	456

7	100	212	255	71
	200	633	595	166
	300	1249	1027	298
	400	2076	1626	466

8	100	200	251	74
	200	599	598	167
	300	1226	1060	292
	400	2021	1581	463
	500	3057	2279	665

Tested on a 64-bit Linux server with 2.13 GHz Intel Core 2 Duo CPU, 2 GB cache, and 16 GB memory.

### Future work

AnyExpress currently has some limitations as it is based on position matching between tag and reference. Hence it misses exon-spanning tags during the COMBINE process. In the Marioni data, about 4% (or around 1,000) of transcript-matched reads were exon-spanning tags. Although these were not counted in the current version of AnyExpress because of their relatively small representation, we are currently working on developing post-processing modules to rescue these tags.

AnyExpress performs within-platform normalization and quantile normalization [28] for closed-platforms, and the RPKM-like method [29] for an open-platform normalization. However, the current version of AnyExpress does not offer cross-platform normalization. Systematic biases may originate from different platforms, hybridization protocols, time of day when an assay was performed, replicates, and/or amplification reagents. Some investigators have proposed pre-processing methods to remove systematic biases: Singular Value Decomposition (SVD) [42], Distance Weighted Discrimination (DWD) [43], and an empirical Bayes method [44]. However, these methods focus on microarray, not NGS, data and only a small number of arrays are considered. Or, they perform "over-normalization" to the point that biological variations of interest may be lost [44]. NGS technology is still new and a thorough investigation of NGS-specific systematic biases is needed. AnyExpress is modular and open-source, so it is easy to extend and modify. The above-mentioned sources of systematic bias can occur in many of the different analysis steps depicted in Figure 1. However, we implemented AnyExpress in a modular fashion so that users can easily make changes to the current source code to handle the systematic bias in each step. AnyExpress targets an audience with some computational knowledge and hardware with at least 8 GB memory. We have shown that AnyExpress successfully combines 500 .*cel *files and six NGS data. Currently, we are extending AnyExpress in a distributed computing environment to accommodate a larger study.

## Conclusions

We developed AnyExpress, a toolkit that combines and filters cross-platform gene expression data. With sequence-oriented tag mapping and a fast interval algorithm, AnyExpress uniquely offers all of the following features: (i) combine cross-platform gene expression data at a user-defined gene expression unit level (gene, isoform, or exon), (ii) process gene expression data from both open- and closed-platforms, (iii) select a preferred custom target reference, (iv) exclude undesirable tags based on custom-defined biological features, (v) create a coverage plot along the genomic regions of interest, (vi) bind a large number of Affymetrix .*cel *files into a single text file, and (vii) perform quantile-normalization with a large number of microarray samples.

## Availability and requirements

• Project name: AnyExpress

• Project home page: http://anyexpress.sourceforge.net

• Operating system: Linux, Unix, Mac OS X, or Windows

• Programming language: Java, shell script, and Python

• License: Apache License version 2.0

## List of abbreviations

BED: Browser Extensible Data; CARD: Catalysed Reporter Deposition; CAT: Correspondence At the Top; CC: Correlation coefficient; DD: Differential Display; DWD: Distance Weighted Discrimination; FDR: False Discovery Rate; FISH: Fluorescent In Situ Hybridization; GEO: Gene Expression Omnibus; ID: Identifier; MAQC: Microarray Quality Control; MPSS: Massively Parallel Signature Sequencing; NGS: Next-Generation Sequencing; RMA: Robust Multi-array Averaging; RPKM: Reads Per Kilobase exon model per Million mapped reads; SAGE: Serial Analysis of Gene Expression; SD: Standard Deviation; SNP: Single Nucleotide Polymorphism; SVD: Singular Value Decomposition

## Authors' contributions

JK initiated the project, designed and implemented the software, and drafted the paper. KP architected the software and implemented core modules. HJ processed data, performed testing, and generated figures/tables. WPK and LOM conceived the study and designed and directed the project. All co-authors contributed to manuscript preparation.

## Supplementary Material

Additional file 1 **AT plot based on statistical significance**. This CAT plot depicts the agreement between NGS and microarray data on the detection of differently expressed genes. The X-axis is the number of top genes in NGS data, ranked by the statistical significance (FDR adjusted q-value) of kidney vs. liver. The Y-axis is the proportion of genes from the microarray that is in common with top-ranked genes from NGS. Four CAT plots are drawn using none, snp, multiTarget, and both (snp and multiTarget simultaneously) exclusion features. Click here for file

## References

[B1] Barrett T Troup DB Wilhite SE Ledoux P Rudnev D Evangelista C Kim IF Soboleva A Tomashevsky M Edgar R NCBI GEO: mining tens of millions of expression profiles--database and tools update Nucleic Acids Res 2007 35 Database D760 765 10.1093/nar/gkl887 17099226 PMC1669752

[B2] Ramasamy A Mondry A Holmes CC Altman DG Key Issues in Conducting a Meta-Analysis of Gene Expression Microarray Datasets PLoS Med 2008 5 9 e184 10.1371/journal.pmed.0050184 18767902 PMC2528050

[B3] Hong F Breitling R A comparison of meta-analysis methods for detecting differentially expressed genes in microarray experiments Bioinformatics 2008 24 3 374 382 10.1093/bioinformatics/btm620 18204063

[B4] Rhodes DR Barrette TR Rubin MA Ghosh D Chinnaiyan AM Meta-analysis of microarrays: interstudy validation of gene expression profiles reveals pathway dysregulation in prostate cancer Cancer Res 2002 62 15 4427 4433 12154050

[B5] Warnat P Eils R Brors B Cross-platform analysis of cancer microarray data improves gene expression based classification of phenotypes BMC Bioinformatics 2005 6 265 10.1186/1471-2105-6-265 16271137 PMC1312314

[B6] Dai M Wang P Boyd AD Kostov G Athey B Jones EG Bunney WE Myers RM Speed TP Akil H Evolving gene/transcript definitions significantly alter the interpretation of GeneChip data Nucleic Acids Res 2005 33 20 e175 10.1093/nar/gni179 16284200 PMC1283542

[B7] Mecham BH Klus GT Strovel J Augustus M Byrne D Bozso P Wetmore DZ Mariani TJ Kohane IS Szallasi Z Sequence-matched probes produce increased cross-platform consistency and more reproducible biological results in microarray-based gene expression measurements Nucleic Acids Res 2004 32 9 e74 10.1093/nar/gnh071 15161944 PMC419626

[B8] Benovoy D Kwan T Majewski J Effect of polymorphisms within probe-target sequences on olignonucleotide microarray experiments Nucleic Acids Res 2008 36 13 4417 4423 10.1093/nar/gkn409 18596082 PMC2490733

[B9] Sandberg R Larsson O Improved precision and accuracy for microarrays using updated probe set definitions BMC Bioinformatics 2007 8 48 10.1186/1471-2105-8-48 17288599 PMC1805763

[B10] Kong SW Hwang KB Kim RD Zhang BT Greenberg SA Kohane IS Park PJ CrossChip: a system supporting comparative analysis of different generations of Affymetrix arrays Bioinformatics 2005 21 9 2116 2117 10.1093/bioinformatics/bti288 15684227 PMC2819168

[B11] Yi Y Li C Miller C George AL Jr Strategy for encoding and comparison of gene expression signatures Genome Biol 2007 8 7 R133 10.1186/gb-2007-8-7-r133 17612401 PMC2323223

[B12] Lacson R Pitzer E Hinske C Galante P Ohno-Machado L Evaluation of a large-scale biomedical data annotation initiative BMC Bioinformatics 2009 10 Suppl 9 S10 10.1186/1471-2105-10-S9-S10 19761564 PMC2745681

[B13] Bisognin A Coppe A Ferrari F Risso D Romualdi C Bicciato S Bortoluzzi S A-MADMAN: annotation-based microarray data meta-analysis tool BMC Bioinformatics 2009 10 201 10.1186/1471-2105-10-201 19563634 PMC2711946

[B14] Zhou X Su Z Sammons RD Peng Y Tranel PJ Stewart CN Yuan JS Novel software package for cross-platform transcriptome analysis (CPTRA) BMC Bioinformatics 2009 10 Suppl 11 S16 10.1186/1471-2105-10-S11-S16 19811681 PMC3226187

[B15] Kuo WP Liu F Trimarchi J Punzo C Lombardi M Sarang J Whipple ME Maysuria M Serikawa K Lee SY A sequence-oriented comparison of gene expression measurements across different hybridization-based technologies Nat Biotechnol 2006 24 7 832 840 10.1038/nbt1217 16823376

[B16] Liu F Jenssen TK Trimarchi J Punzo C Cepko CL Ohno-Machado L Hovig E Kuo WP Comparison of hybridization-based and sequencing-based gene expression technologies on biological replicates BMC Genomics 2007 8 153 10.1186/1471-2164-8-153 17555589 PMC1899500

[B17] Shi L Reid LH Jones WD Shippy R Warrington JA Baker SC Collins PJ de Longueville F Kawasaki ES Lee KY The MicroArray Quality Control (MAQC) project shows inter- and intraplatform reproducibility of gene expression measurements Nat Biotechnol 2006 24 9 1151 1161 10.1038/nbt1239 16964229 PMC3272078

[B18] Lacson R Pitzer E Kim J Galante P Hinske C Ohno-Machado L DSGeo: Software tools for cross-platform analysis of gene expression data in GEO J Biomed Inform 2010 20435161 10.1016/j.jbi.2010.04.007 PMC2934864

[B19] Kim J Pitzer E Galante P Hinske C Kuo WP Lacson R Ohno-Machado L ExpressionCombiner:a web-based tool for cross-platform analysis of gene expression data Am Med Informatics Assoc Summit Translational Bioinformatics 2009 S08

[B20] Pitzer E Kim J Patel K Galante PA Ohno-Machado. L PositionMatcher: A Fast Custom-Annotation Tool for Short DNA Sequences Am Med Informatics Assoc Summit Translational Bioinformatics 2010 S22 PMC3041550 21347141

[B21] Sukardi H Ung CY Gong Z Lam SH Incorporating zebrafish omics into chemical biology and toxicology Zebrafish 2010 7 1 41 52 10.1089/zeb.2009.0636 20384484

[B22] Vieites JM Guazzaroni ME Beloqui A Golyshin PN Ferrer M Metagenomics approaches in systems microbiology FEMS Microbiol Rev 2009 33 1 236 255 10.1111/j.1574-6976.2008.00152.x 19054115

[B23] Langmead B Trapnell C Pop M Salzberg SL Ultrafast and memory-efficient alignment of short DNA sequences to the human genome Genome Biol 2009 10 3 R25 10.1186/gb-2009-10-3-r25 19261174 PMC2690996

[B24] Sherry ST Ward MH Kholodov M Baker J Phan L Smigielski EM Sirotkin K dbSNP: the NCBI database of genetic variation Nucleic Acids Res 2001 29 1 308 311 10.1093/nar/29.1.308 11125122 PMC29783

[B25] Thompson KJ Deshmukh H Solka JL Weller JW A white-box approach to microarray probe response characterization: the BaFL pipeline BMC Bioinformatics 2009 10 449 10.1186/1471-2105-10-449 20040098 PMC2804686

[B26] Ferrari F Bortoluzzi S Coppe A Sirota A Safran M Shmoish M Ferrari S Lancet D Danieli GA Bicciato S Novel definition files for human GeneChips based on GeneAnnot BMC Bioinformatics 2007 8 446 10.1186/1471-2105-8-446 18005434 PMC2216044

[B27] Marioni JC Mason CE Mane SM Stephens M Gilad Y RNA-seq: an assessment of technical reproducibility and comparison with gene expression arrays Genome Res 2008 18 9 1509 1517 10.1101/gr.079558.108 18550803 PMC2527709

[B28] Irizarry RA Hobbs B Collin F Beazer-Barclay YD Antonellis KJ Scherf U Speed TP Exploration, normalization, and summaries of high density oligonucleotide array probe level data Biostatistics 2003 4 2 249 264 10.1093/biostatistics/4.2.249 12925520

[B29] Mortazavi A Williams BA McCue K Schaeffer L Wold B Mapping and quantifying mammalian transcriptomes by RNA-Seq Nat Methods 2008 5 7 621 628 10.1038/nmeth.1226 18516045 PMC13303166

[B30] Bhattacharjee A Richards WG Staunton J Li C Monti S Vasa P Ladd C Beheshti J Bueno R Gillette M Classification of human lung carcinomas by mRNA expression profiling reveals distinct adenocarcinoma subclasses Proc Natl Acad Sci USA 2001 98 24 13790 13795 10.1073/pnas.191502998 11707567 PMC61120

[B31] Sotiriou C Wirapati P Loi S Harris A Fox S Smeds J Nordgren H Farmer P Praz V Haibe-Kains B Gene expression profiling in breast cancer: understanding the molecular basis of histologic grade to improve prognosis J Natl Cancer Inst 2006 98 4 262 272 10.1093/jnci/djj052 16478745

[B32] Schmidberger M Vicedo E Mansmann U affyPara-a Bioconductor Package for Parallelized Preprocessing Algorithms of Affymetrix Microarray Data Bioinform Biol Insights 2009 3 83 87 20140068 10.4137/bbi.s3060 PMC2808179

[B33] Bolstad BM Irizarry RA Astrand M Speed TP A comparison of normalization methods for high density oligonucleotide array data based on variance and bias Bioinformatics 2003 19 2 185 193 10.1093/bioinformatics/19.2.185 12538238

[B34] Huang W Marth G EagleView: a genome assembly viewer for next-generation sequencing technologies Genome Res 2008 18 9 1538 1543 10.1101/gr.076067.108 18550804 PMC2527701

[B35] Aho AV Kernighan BW Weinberger PJ The AWK programming language 1988 Reading, Mass.: Addison-Wesley Pub. Co

[B36] Subramanian A Tamayo P Mootha VK Mukherjee S Ebert BL Gillette MA Paulovich A Pomeroy SL Golub TR Lander ES Gene set enrichment analysis: a knowledge-based approach for interpreting genome-wide expression profiles Proc Natl Acad Sci USA 2005 102 43 15545 15550 10.1073/pnas.0506580102 16199517 PMC1239896

[B37] Irizarry RA Warren D Spencer F Kim IF Biswal S Frank BC Gabrielson E Garcia JG Geoghegan J Germino G Multiple-laboratory comparison of microarray platforms Nat Methods 2005 2 5 345 350 10.1038/nmeth756 15846361

[B38] Ottaviano FG Tang SS Handy DE Loscalzo J Regulation of the extracellular antioxidant selenoprotein plasma glutathione peroxidase (GPx-3) in mammalian cells Mol Cell Biochem 2009 327 1-2 111 126 10.1007/s11010-009-0049-x 19219623 PMC2693281

[B39] Daniel VC Marchionni L Hierman JS Rhodes JT Devereux WL Rudin CM Yung R Parmigiani G Dorsch M Peacock CD A primary xenograft model of small-cell lung cancer reveals irreversible changes in gene expression imposed by culture in vitro Cancer Res 2009 69 8 3364 3373 10.1158/0008-5472.CAN-08-4210 19351829 PMC2821899

[B40] Laubinger S Zeller G Henz SR Sachsenberg T Widmer CK Naouar N Vuylsteke M Scholkopf B Ratsch G Weigel D At-TAX: a whole genome tiling array resource for developmental expression analysis and transcript identification in Arabidopsis thaliana Genome Biol 2008 9 7 R112 10.1186/gb-2008-9-7-r112 18613972 PMC2530869

[B41] Storey JD Tibshirani R Statistical significance for genomewide studies Proc Natl Acad Sci USA 2003 100 16 9440 9445 10.1073/pnas.1530509100 12883005 PMC170937

[B42] Alter O Brown PO Botstein D Singular value decomposition for genome-wide expression data processing and modeling Proc Natl Acad Sci USA 2000 97 18 10101 10106 10.1073/pnas.97.18.10101 10963673 PMC27718

[B43] Benito M Parker J Du Q Wu J Xiang D Perou CM Marron JS Adjustment of systematic microarray data biases Bioinformatics 2004 20 1 105 114 10.1093/bioinformatics/btg385 14693816

[B44] Johnson WE Li C Rabinovic A Adjusting batch effects in microarray expression data using empirical Bayes methods Biostatistics 2007 8 1 118 127 10.1093/biostatistics/kxj037 16632515

