# Supplementary figures and images for: AnyExpress: Integrated toolkit for analysis of cross-platform gene expression data using a fast interval matching algorithm
Source: BMC Bioinformatics. 2011 Mar 17;12:75. doi: 10.1186/1471-2105-12-75 (PMC3076267; doi:10.1186/1471-2105-12-75)

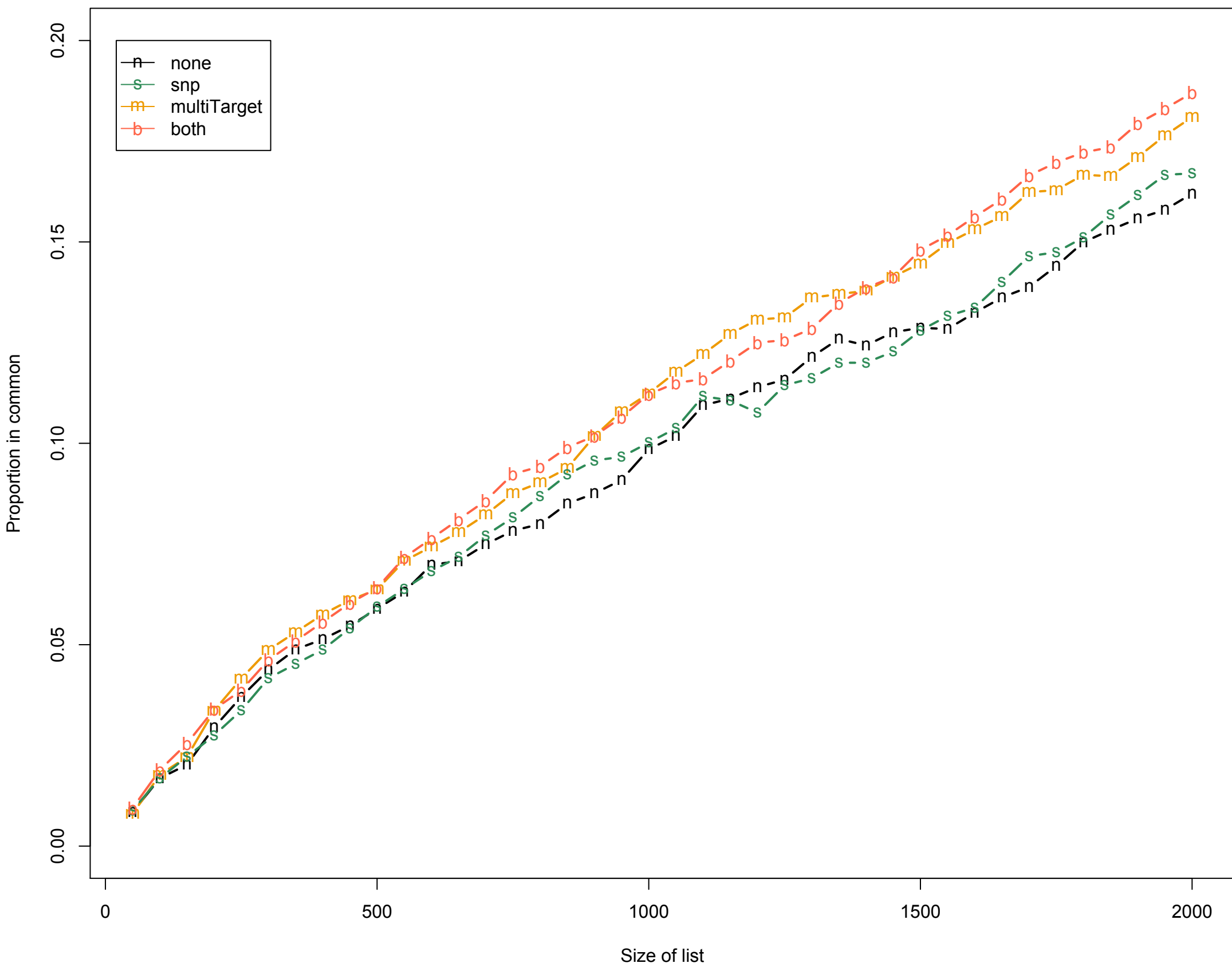

Supplement: Additional file 1 — AT plot based on statistical significance. This CAT plot depicts the agreement between NGS and microarray data on the detection of differently expressed genes. The X-axis is the number of top genes in NGS data, ranked by the statistical significance (FDR adjusted q-value) of kidney vs. liver. The Y-axis is the proportion of genes from the microarray that is in common with top-ranked genes from NGS. Four CAT plots are drawn using none, snp, multiTarget, and both (snp and multiTarget simultaneously) exclusion features. [file 1471-2105-12-75-S1.PDF]
